# Functional tic-like presentations differ strikingly from Provisional Tic Disorder

**DOI:** 10.12688/f1000research.129252.1

**Published:** 2022-12-22

**Authors:** Amanda L. Arbuckle, Emily C. Bihun, Bradley L. Schlaggar, Kevin J. Black

**Affiliations:** 1 Department of Psychiatry, Washington University in St. Louis, School of Medicine, St. Louis, Missouri, 63110, USA; 2 Kennedy Krieger Institute, Baltimore, Maryland, 21205, USA; 3 Department of Neurology, Johns Hopkins Medicine, Baltimore, Maryland, 21287, USA; 4 Department of Pediatrics, Johns Hopkins Medicine, Baltimore, MD, 21287, USA; 5 Department of Neurology, Washington University in St. Louis, School of Medicine, St. Louis, Missouri, 63110, USA; 6 Department of Radiology, Washington University in St. Louis, School of Medicine, St. Louis, Missouri, 63110, USA; 7 Department of Neuroscience, Washington University in St. Louis, School of Medicine, St. Louis, Missouri, 63110, USA

**Keywords:** Tic Disorders/classification, Provisional Tic Disorder, Functional Neurological Symptom Disorder, Conversion Disorder, Diagnosis, Differential, Tourette Syndrome

## Abstract

**Background:** Recent years have seen a dramatic increase in new “tic” cases in teens and young adults. These individuals often present with fulminant onset of symptoms not commonly seen in Tourette syndrome (TS) and are often diagnosed with Functional Neurological Symptom Disorder (FND-tic). However, some authors have questioned whether this illness truly differs from typical Provisional Tic Disorder (PTD) and TS. Previous studies have compared FND-tic, usually a few months after symptom onset, to patients with TS, usually years after symptom onset. We sought to test whether the presenting symptoms of FND-tic differ substantially from those in patients at a similar duration of symptoms who are later diagnosed with TS.

**Methods:** This comparative study examines clinical features summarized from published reports of FND-tic with novel data from a longitudinal study of PTD. This study came from a referral center for TS and tic disorders and included 89 children with tics whose first tic occurred a median of 3.6 months earlier, nearly all of whom were diagnosed with a chronic tic disorder at follow-up. Specifically, we examine clinical features identified in a recent literature review as supporting a diagnosis of FND-tic, including symptom characteristics, course, severity and comorbidity.

**Results:** Several clinical features dramatically distinguish the patients diagnosed with FND-tic from those diagnosed with typical PTD. For example, coprophenomena are reported at or shortly after symptom onset in over half of FND-tic patients, whereas even several months after onset, coprophenomena had occurred in only 1 of 89 children with PTD. Six clinical features each have a positive predictive value over 90% for FND-tic diagnosis if prior probability is 50%.

**Conclusions: **These new data provide strong evidence supporting the diagnostic validity of FND-tic as distinct from TS.

## Introduction

Recent years have seen a dramatic increase in new “tic” cases in teens and young adults. ^1^^,^^2^ These individuals often present with fulminant onset of symptoms not commonly seen in Tourette syndrome (TS), but often similar to those found in videos tagged as “Tourette” on social media. ^3^ For instance, echopraxia and coprolalia occur in more than half of these individuals at symptom onset. ^3^ The nature and characteristics of these symptoms, and the onset age and course of illness have led experienced clinicians to differentiate these cases from TS and to diagnose instead Functional Neurological Symptom Disorder (FND). FND with tic-like symptoms (hereinafter “FND-tic”) has been reported previously, but prior to 2019 was considered rare, occurring in < 2% of tic or tic-like cases at five major referral centers from three continents. ^4^

Some experts have expressed skepticism as to whether a new diagnosis (FND) is needed for these patients, positing that the previous understanding of TS may have been too narrow. ^5^ Perhaps, for instance, echopraxia is present early in the course of TS much more often than has been recognized. We concur with these authors that clearly differentiating a new diagnosis from existing diagnoses is a key component of diagnostic validity. ^6^ Substantial published data describe typical clinical features of chronic tic disorders, but almost no prospective data have been published on symptoms in the first year after tic onset. ^7^^,^^8^ This evidence gap is crucial because most FND-tic patients at clinical centers have had symptoms for only a few months (mean 0.4 years in one study ^4^). Here, we directly address this concern by providing for the first time substantial data on relevant clinical features during the first few months after symptom onset in children ultimately diagnosed with TS.

## Methods

The New Tics study is a prospective, longitudinal study that enrolled 89 children ages 5–10 years whose first tic occurred in the past 9 months (median 3.6 months). ^9^ Children are assessed using multiple informants (child, parent, trained interviewer, and observation by an experienced clinician for more than an hour, including by video while the child is alone). The diagnosis in this situation is Provisional Tic Disorder (PTD), and nearly all these children (77 of 79) were diagnosed with TS (70) or a persistent tic disorder (7) when they returned at the one-year anniversary of their first tic. ^10^ Here we report the prevalence and timing from the New Tics sample of various features that occur commonly in FND-tic patients.

The feature list was drawn from a recent review of FND-tic, a narrative review that included all primary data publications on FND-tic known to its authors as of August, 2022. ^3^ The comparison data for FND-tic patients comes from 26 published reports, with pertinent data in 17 reports ^4^^,^^11^^–^^26^ describing a total of 336 patients (data file available as *Underlying data* ^27^). For quantitative variables, the weighted mean is provided (weighted by *N* in each report), along with the median and range of the summary values reported in each relevant publication.

Most of these features thought to suggest FND-tic were recorded prospectively in the New Tics study, including age, sex, premonitory urges, tic suppression, coprophenomena, and family history. However, since the New Tics sample was enrolled almost entirely before the recent FND-tic upsurge, some of these clinical features were recorded indirectly. For instance, to match “severe symptoms at onset,” defined differently in various reports on FND-tic, we conservatively chose from the New Tics sample all patients with emergency department visits or disability prior to the screening visit, or a high score on the Yale Global Tic Severity Scale’s ^28^ impairment item at screening. Details on other such choices are given in footnotes to Table 1.

**Table 1.** Comparison of various clinical features in FND-tic and in typical PTD. ^a^ FND, Functional Neurological Symptom Disorder; FND-tic, Functional Neurological Symptom Disorder with tic-like symptoms; PTD, Provisional Tic Disorder; NP, number of publications; OCD, obsessive compulsive disorder; ADHD, attention deficit hyperactivity disorder; YGTSS, Yale Global Tic Severity Scale.

Feature	FND NP ^b^	FND numerator/denominator (percentage)	FND median percentage (range)	New Tics ^c^ number (percentage)
Sex (% female)	17	251/336 (75)	72 (20–100)	25 (28)
Typical tic disorder diagnosis prior to current episode onset	11	33/150 (22)	15 (0–100)	21/125 (17) ^d^
Sudden, abrupt onset	10 ^e^	136/142 (96)	100 (77–100)	15/75 (20) ^f^ or 46/62 (74) ^g^
Symptoms in extremities before face and neck	7	43/100 (43)	40 (15–100)	10 (11)
Coprophenomena at onset	8	68/115 (59)	54 (0–77)	1 (1)
Tics involving the body or limbs without a history of tics involving the eyes, face, and head	7	29/84 (35)	18 (0–77)	10 (11)
Premonitory urges present	9	56/118 (48)	60 (0–100)	85 (96)
Severe symptoms at onset	6	82/172 (48)	77 (30–100)	3–5 (3–6)
Extreme “attacks” of tic-like behavior	4	44/68 (65)	82 (36–100)	0–2 (0–2)
Inability to suppress	10	74/120 (62)	70 (0–100)	20 (22)
Tic-like phenomena are constant in severity over time rather than waxing and waning	5	50/75 (67)	68 (15–100)	51 (58)
Movements or vocalizations that are dramatically worse in the presence of others *versus* when alone	3	15/32 (47)	50 (11–100)	0 (0) ^h^
Symptoms that dramatically and persistently disrupt the person’s intended actions or communications	3	34/52 (65)	39 (36–89)	2 (2)
Family history of tics	9	17/131 (13)	0 (0–60)	30 (34)
Family history of OCD	1	1/22 (5)	5	14 (16)
Family history of ADHD	1	6/22 (27)	27	25 (28)
ADHD diagnosis before/at presentation	9	69/216 (32)	22 (0–48)	39 (43)
OCD diagnosis before/at presentation	8	11/127 (9)	6 (0–23)	27 (30)
Anxiety disorder prior to/at presentation	8	77/132 (58)	53 (11–100)	27 (30)

Feature	FND NP	FND weighted mean	FND median (range)	New Tics mean
Age of onset	15	22.3	16.5 (7.5–53.6)	7.6
Age at presentation	5	20.5	18.8 (11.2–36.3)	7.9
YGTSS ^i^ Total Tic Score (0-50)	2	32.7	32.4 (31.5–33.3)	16.9
YGTSS Impairment (0-50)	2	30.2	31.2 (28.6–33.8)	7.6
YGTSS Global Severity Score (0-100)	3	62.8	62.6 (61.9–65.3)	24.3

^a^ Specified clinical features in patients with tic-like symptoms (“FND”) from the articles reviewed in Malaty *et al.* (2022), ^3^ compared to participants with typical Provisional Tic Disorder from the New Tics study (“New Tics”).

^b^ NP = number of publications from the table in Malaty *et al.* (2022) ^3^ that contributed data to the statistics in this row.

^c^ N = 89 except where numerator and denominator are provided.

^d^ N = 125, the number of participants who came for a screening visit after reporting recent onset of tics during initial telephone contact. Some were found during careful screening to have a prior episode of transient tics.

^e^ Excludes the two reports that defined the sample by sudden onset. ^4^^,^^21^

^f^ The investigator assigned the most likely symptom onset date within a date range (possible onset dates) of less than 7 days.

^g^ From parents’ answers as to whether the onset of the first tic was sudden or gradual.

^h^ Investigator noted tics during the history and exam (approximately 45 minutes), but no tics when observing the child alone in the room via video camera (approximately 40 minutes).

^i^ Yale Global Tic Severity Scale.

### Data analysis

Fisher’s exact test was used to find the probability of differences in frequency of features between the two populations (fisher_exact from SciPy (RRID:SCR_008058) 1.9.1). ^29^

### Ethical considerations

The New Tics study was approved by the Washington University Human Research Protection Office (IRB, protocol numbers 201109157 and 201707059), all participants assented to participation, and a parent or other legal guardian provided written documentation of informed consent.

## Results

Stark differences in presentation distinguish the FND-tic patients from typical PTD ( Table 1). For example, coprophenomena are reported at or shortly after symptom onset in 59% of FND-tic patients. By contrast, coprophenomena had occurred in only 1 of 89 children with PTD at an average of 3.6 months after tic onset. Similarly, the TS International Database Consortium found that only 2% of TS patients in tertiary centers retrospectively reported coprophenomena at symptom onset, and only 20% ever manifested coprophenomena by an average of 5 years after tic onset. ^30^ Movements or vocalizations that were dramatically worse in the presence of others *versus* when alone occurred in 47% of FND-tic patients, but in none of the New Tics PTD sample. Symptoms dramatically and persistently disrupted intended actions in 65% of FND-tic patients, but in only 2% of PTD. The prevalence of prolonged tic attacks was 65% in FND-tic, but 0–2% in PTD. Other features that differed substantially include lack of premonitory urges (53% *vs.* 4%) and severe symptoms at onset (48% *vs.* 3–6%). Table 1 and Table 2 provide details on these comparisons and include statistics on a dozen more clinical features of FND-tic that differ from the New Tics PTD sample.

In a patient with recent onset of tics, the presence of any one of the features named above raises the probability of a non-TS diagnosis from 50% (as when the clinician is ambivalent about the diagnosis prior to considering this feature) to over 90% ( Table 2). Other features differ significantly but are less useful diagnostically. For instance, obsessive compulsive disorder (OCD) is more than three times less common in FND-tic than in PTD *(p*<.0001) *,* but its absence only raises the probability of FND-tic from 50% to 57%.

**Table 2.** Diagnostic utility of the binary features in Table 1. ^a^ PPV, positive predictive value; NPV, negative predictive value; OCD, obsessive compulsive disorder; ADHD, attention deficit hyperactivity disorder; TS, Tourette syndrome; FND, Functional Neurological Symptom Disorder.

Feature	*p* ^b^	PPV (NPV)	prior 2%	prior 50%
Movements or vocalizations that are dramatically worse in the presence of others *versus* when alone	**.000**	PPV =	**100%**	**100%**
Coprophenomena at onset	**.000**	PPV =	52%	**98%**
Coprolalia at presentation	**.000**	PPV =	47%	**98%**
Symptoms that dramatically and persistently disrupt the person’s intended actions or communications	**.000**	PPV =	38%	**97%**
Extreme “attacks” of tic-like behavior	**.000**	PPV =	38%	**97%**
Premonitory urges present	**.000**	**NPV =**	19%	**92%**
Severe symptoms at onset (defined variously in different studies)	**.000**	PPV =	15%	**89%**
Sudden, abrupt onset (NewTics: onset confidence window < 7 days)	**.000**	PPV =	9%	**83%**
Symptoms in extremities before face and neck	**.000**	PPV =	7%	**79%**
Tics involving the body or limbs without a history of tics involving the eyes, face, and head	**.000**	PPV =	6%	**75%**
Inability to suppress	**.000**	PPV =	5%	**73%**
Female	**.000**	PPV =	5%	**73%**
Anxiety disorder prior to/at presentation	**.000**	PPV =	4%	66%
Family history of tics	**.000**	**NPV =**	3%	57%
OCD diagnosis before/at presentation	**.000**	**NPV =**	3%	57%
Typical tic disorder diagnosis prior to current episode onset	.185	PPV =	3%	57%
Sudden, abrupt onset (NewTics: per parent tic survey)	**.000**	PPV =	3%	56%
ADHD diagnosis before/at presentation	.050	**NPV =**	2%	54%
Tic-like phenomena are constant in severity over time rather than waxing and waning	.164	PPV =	2%	53%
Family history of OCD	.152	**NPV =**	2%	53%
Family history of ADHD	.584	**NPV =**	2%	50%

^a^ PPV of a non-TS diagnosis for the binary features in Table 1, assuming a prior probability for FND of 2% (typical pre-pandemic prevalence at a referral center ^4^) or 50% (representing clinical equipoise about a given patient’s diagnosis before considering this feature). NPV is shown for features more common in typical TS, equivalent to PPV for the absence of the given feature.

^b^ Fisher’s exact test; “.000” means < .0005; *p* values bolded if *p* < .05/20 = .0025.

Note: Values for PPV and NPV are bolded if ≥67%.

## Discussion

We demonstrate conclusively that patients with functional tic-like symptoms differ notably from typical tic patients at the same stage of the disorder, namely in the first few months after symptom onset. Previous reports have compared FND-tic to TS, ^3^^,^^31^ but not to a large PTD sample. The older age of the FND-tic group could be seen as a limitation of our study, since one might posit different symptoms at different ages of tic onset might be part of the natural history of tic disorder. However, this theoretical concern does not in fact limit our conclusions, because retrospective studies of TS and a prospective study of PTD in siblings of TS probands all found peak tic onset before age 10 years old in both sexes; adult onset of tics is uncommon. ^7^^,^^8^^,^^32^

The data presented here do not prove the etiology of the tic-like symptoms diagnosed in the cited reports; hence the limited claim that these symptoms represent a different illness than PTD/TS. However, the marked difference in presentation these data demonstrate is an important argument adduced in the cited reports to support the diagnosis of functional neurological symptom disorder. Diagnosing FND-tic is important, since to the extent of our current knowledge, its prognosis and optimal treatment differ from those of TS. ^3^

In conclusion, these new clinical data about the first few months after tic onset prior to diagnosis of TS provide strong evidence supporting the diagnostic validity of functional tic-like symptoms as distinct from PTD and TS.

## Data Availability

Information on ‘The New Tics Study: A Novel Approach to Pathophysiology and Cause of Tic Disorders’ can be found at NIMH Data Archive and NIH RePORTER (Project Number 1R01MH104030-01A1). Open Science Framework: Supplemental materials for publication: Functional tic-like presentations differ strikingly from Provisional Tic Disorder. https://doi.org/10.17605/OSF.IO/RSFXN ^27^. This project contains the following underlying data:
-
FNS_vs_TS.csv (summary table from the FND-tic publications cited by Malaty et al. ^3^) - FND-tic_not_PTD.py (python script used to summarize data for Tables 1 and 2) Data are available under the terms of the Creative Commons Attribution 4.0 International license (CC-BY 4.0).
