# Supplementary material for: Functional tic-like presentations differ strikingly from Provisional Tic Disorder
Source: F1000Res. 2023 Apr 17;11:1566. Originally published 2022 Dec 22. [Version 2] doi: 10.12688/f1000research.129252.2 (PMC10186060; doi:10.12688/f1000research.129252.2)
Supplement: Peer review history

# Peer review: Reviewer response for version 2

**DOI:** 10.5256/f1000research.146566.r169890

**Published:** 2023-05-15

**Authors:** Michael Bloch

**Affiliations:** 1 Department of Psychiatry, Yale School of Medicine, New Haven, Connecticut, USA

The authors have responded adequately to my critiques. I appreciate this important contribution to the study of tic disorders.

Is the work clearly and accurately presented and does it cite the current literature?

Yes

If applicable, is the statistical analysis and its interpretation appropriate?

Yes

Are all the source data underlying the results available to ensure full reproducibility?

Yes

Is the study design appropriate and is the work technically sound?

Yes

Are the conclusions drawn adequately supported by the results?

Partly

Are sufficient details of methods and analysis provided to allow replication by others?

Yes

Reviewer Expertise:

Meta-analysis, psychopharmacology, tic disorders, OCD

I confirm that I have read this submission and believe that I have an appropriate level of expertise to confirm that it is of an acceptable scientific standard.

# Peer review: Reviewer response for version 2

**DOI:** 10.5256/f1000research.146566.r169889

**Published:** 2023-04-18

**Authors:** Tammy Hedderly

**Affiliations:** 1 Paediatric Neurology Department, Evelina London Children’s Hospital, King's College London, Guy’s & St Thomas’ NHS Foundation Trust, London, England, UK

Thank you to the authors for their response to all my queries and for the clarification. I would like to approve this article as a helpful addition to this field.

Is the work clearly and accurately presented and does it cite the current literature?

Yes

If applicable, is the statistical analysis and its interpretation appropriate?

Partly

Are all the source data underlying the results available to ensure full reproducibility?

Yes

Is the study design appropriate and is the work technically sound?

Yes

Are the conclusions drawn adequately supported by the results?

Partly

Are sufficient details of methods and analysis provided to allow replication by others?

Yes

Reviewer Expertise:

Paediatric Neurology , Movement Disorders and Tourettes

I confirm that I have read this submission and believe that I have an appropriate level of expertise to confirm that it is of an acceptable scientific standard.

# Peer review: Reviewer response for version 1

**DOI:** 10.5256/f1000research.141921.r158571

**Published:** 2023-03-31

**Authors:** Tammy Hedderly

**Affiliations:** 1 Paediatric Neurology Department, Evelina London Children’s Hospital, King's College London, Guy’s & St Thomas’ NHS Foundation Trust, London, England, UK

The authors, all highly experienced 'Tourettologists', have approached this topic in an interesting and novel manner to try and provide some evidence for the ability to differentiate functional tic like behaviours from those seen in provisional tic disorders, usually related to Tourettes at follow up. This is a complex area, with some researchers proposing some shared mechanisms and overlapping neurobiology in the presentations. This paper uses some usefully collected detailed (although some retrospective) clinical data of children with PTD within a few months of presentation to compare characteristics demonstrating some useful distinguishing features. The presentation of the statistics could benefit from a little more expansion and explanation, especially for the non-expert, as it seems an important aspect of this paper. For example, table 2 is not that clearly explained and could do with more detail.

 There are some limitations, but these have been clearly highlighted by the authors, such as the difference in ages of the two groups. I therefore would support this paper as it represents a useful contribution to the ongoing exploration of the functional tic-like phenomenon. I do wonder whether the dichotomy of subjects is a problem and would welcome wider discussions by the authors, on the situations seen commonly in the clinic in which young people, demonstrate both Tourette-related tics and functional tic-like behaviours together. This paper raised a question in my mind, as to whether the authors could have included distinguishing factors for the concept of provisional or transient functional disorder, especially when appearing in the context of exposure to social media or influencers.

 Thank you for an interesting contribution.

Is the work clearly and accurately presented and does it cite the current literature?

Yes

If applicable, is the statistical analysis and its interpretation appropriate?

Partly

Are all the source data underlying the results available to ensure full reproducibility?

Yes

Is the study design appropriate and is the work technically sound?

Yes

Are the conclusions drawn adequately supported by the results?

Partly

Are sufficient details of methods and analysis provided to allow replication by others?

Yes

Reviewer Expertise:

Paediatric Neurology , Movement Disorders and Tourettes

I confirm that I have read this submission and believe that I have an appropriate level of expertise to confirm that it is of an acceptable scientific standard.

# Response

**Published:** 2023-04-06

**Authors:** Kevin J Black

**Affiliations:** Washington University in St Louis, USA

We thank Dr. Hedderly for her thoughtful and expert critique. Below we respond to each comment in turn.

 1. *The presentation of the statistics could benefit from a little more expansion and explanation, especially for the non-expert, as it seems an important aspect of this paper. For example, table 2 is not that clearly explained and could do with more detail.*

 Thank you. We agree. The new text in 'Results' reads as follows:

 "Table 2 illustrates the clinical value of these comparisons, viewing each clinical feature as a “test” for FND-tic, with its associated sensitivity and specificity. We also provide the positive predictive value (PPV) for each feature thought to indicate FND-tic. The PPV represents how confident one can be of a FND-tic diagnosis given the presence of the listed feature. The PPV depends on the prior probability of the FND-tic diagnosis, or its likelihood before one knows whether a given patient has that feature. We provide PPV for each feature based on two prior probabilities, 2% and 50%. The first, 2%, represents the approximate rate before the pandemic of functional tic-like symptoms at clinical referral centers. 4  Clinical equipoise about a given participant’s diagnosis is represented by a prior probability of 50%, for instance if one knows that a referring clinician is ambivalent about whether the patient has PTD or FND-tic, but one has not yet read the chart nor seen the patient. Features thought to be less common in FND-tic are listed with a negative predictive value (NPV), equivalent to PPV for the absence of the given feature."

 2. *I do wonder whether the dichotomy of subjects is a problem and would welcome wider discussions by the authors, on the situations seen commonly in the clinic in which young people, demonstrate both Tourette-related tics and functional tic-like behaviours together.*

 We agree the discussion did not address this issue previously. The discussion now includes this additional paragraph:

 "We note that 22% of the patients reported in the FND-tic papers in fact had a prior history of typical tics, and many of them exhibited both tics and functional tic-like symptoms at the time they presented due to the latter. We have structured our results around whether FND-tic can be diagnosed, not whether PTD can also be diagnosed. In other words, when a patient presents with a typical history for TS but also new symptoms, the features above would allow more confidence in diagnosing FND-tic in addition to TS."

 3. *This paper raised a question in my mind, as to whether the authors could have included distinguishing factors for the concept of provisional or transient functional disorder, especially when appearing in the context of exposure to social media or influencers.*

 The reviewer raises a very interesting question that appears to include two concepts: diagnostic utility of exposure to social media and nosology. 

 a) The exposure to social media or other examples that the new symptoms appear to recapitulate is an important diagnostic clue. We and others have discussed that issue elsewhere (e.g. Frey *et al. *(2022) and Malaty *et al. *(2022)). Unfortunately, we have no prospectively collected data on this question from the PTD group. Anecdotally, I can report that although over a third of our New Tics study participants had a parent or sibling with tics, only once or twice was there any suggestion that the child's first tic may have appeared to be prompted by the family member's similar tic. We have added to the discussion the following paragraph: 

 "Other features in addition to those studied here may also be important for diagnosis. For instance, exposure to tic-like symptoms on social media was a common feature discussed in the papers discussing FND-tic in the past few years. 3  Unfortunately, we have no prospectively collected data from the PTD group on exposure to others with similar symptoms."

 b) Traditionally, functional neurological symptoms have not been diagnosed differently based on duration of the defining symptoms. The closest analogy I can think of is Briquet's syndrome (and its dramatically modified descendants such as DSM-IV somatization disorder), which required not only atypical, medically unexplained symptoms starting before midlife but also numerous symptoms over time, affecting a variety of organ systems. Personally I would not favor creating a new diagnosis to represent "presumed functional symptoms in the first year since onset".

# Peer review: Reviewer response for version 1

**DOI:** 10.5256/f1000research.141921.r158573

**Published:** 2023-01-25

**Authors:** Michael Bloch

**Affiliations:** 1 Department of Psychiatry, Yale School of Medicine, New Haven, Connecticut, USA

This manuscript compares the clinical characteristics of the sample of patients with Functional Neurological Disorder (FND) described in the literature, with a well-characterized sample of Provisional Tic Disorder. Strengths of the manuscript include the clinical importance and novelty of the data as well as the expertise of the authors and the well-written manuscript. Overall, I think this is a manuscript worthy of indexing, although I have several critiques that could potentially improve the manuscript with revision.

1. I do not believe the primary limitation of the manuscript is really acknowledged in the current draft. Mainly that the FND cases are drawn from the existing literature (where there is likely a large degree of reporting bias) versus a Provisional Tic Disorder sample that is much more general. Basically, there is quite a strong possibility that there are other characteristics associated with group difference other than diagnosis.
2. Please consider providing statistical tests in table 1 as well as sensitivity, specificity in table 2.
3. I think in the conclusion the authors should discuss the importance of testing the potentially meaningful differentiators in an independent dataset.
4. It would also be very worthwhile in further studies to use individual participant data to determine the PPV of multiple predictors to engage in a diagnostic distinction.
5. If possible, a histogram exploring age and gender differences between the two groups would be helpful.

Is the work clearly and accurately presented and does it cite the current literature?

Yes

If applicable, is the statistical analysis and its interpretation appropriate?

Yes

Are all the source data underlying the results available to ensure full reproducibility?

Yes

Is the study design appropriate and is the work technically sound?

Yes

Are the conclusions drawn adequately supported by the results?

Partly

Are sufficient details of methods and analysis provided to allow replication by others?

Yes

Reviewer Expertise:

Meta-analysis, psychopharmacology, tic disorders, OCD

I confirm that I have read this submission and believe that I have an appropriate level of expertise to confirm that it is of an acceptable scientific standard, however I have significant reservations, as outlined above.

# Response

**Published:** 2023-04-06

**Authors:** Kevin J Black

**Affiliations:** Washington University in St Louis, USA

We thank Dr. Bloch for his thoughtful and expert critique. Below we respond to each comment in turn.

 1. *I do not believe the primary limitation of the manuscript is really acknowledged in the current draft. Mainly that the FND cases are drawn from the existing literature (where there is likely a large degree of reporting bias) versus a Provisional Tic Disorder sample that is much more general. Basically, there is quite a strong possibility that there are other characteristics associated with group difference other than diagnosis.*

 We agree and have edited the ms. to reflect this point. However, the disparity is smaller than one might think. Here is our new text in the discussion:

 "The primary concern with the validity of these conclusions arises from the different potential sources of ascertainment bias in the two groups. Fortunately, these differences may not be as problematic as one might suppose. The FND-tic group is older, and if one’s information were limited to this study alone, one might posit that the natural history of tic disorder included different early symptoms at different ages of tic onset. However, the literature includes decades of previous clinical information on typical tic disorders. Retrospective studies of TS and a prospective study of PTD in siblings of TS probands all found peak tic onset before age 10 years old; adult onset of tics is quite uncommon. 7 , 8 , 33 The PTD cases were volunteers willing to participate in a rather intensive research study, and were ascertained by a variety of methods (advertising and referrals from clinicians being the most common) rather than by clinical care-seeking. However, at the screening visit for the study, over 60% of them had sought clinical care for the tics or were planning to. The two FND-tic reports from Calgary, Alberta (child and young adult) were from a prospective registry of all patients seen for tics at their center, which is the only specialty center in their region. FND-tic cases were defined by “rapid onset of complex tic-like behaviors, with escalation to peak severity within hours to days.” In other words, these reports are selected only for being seen at a specialty center for tic-like phenomena. Yet their clinical features are similar to those of the FND-tic literature overall (or even more different from the PTD group). For instance, 95% of the Calgary FND-tic child patients were of female sex, only 25% had ADHD, and only 5% had OCD. We can conclude that potential bias from selecting more interesting, severe or classic cases for published case series does not substantially alter our results. Perhaps most importantly, there are no larger published sources for data about FND-tic nor about PTD. Nevertheless, ideally the results presented here should be confirmed in a new, independent sample."

 2. *Please consider providing statistical tests in table 1 as well as sensitivity, specificity in table 2.*

 Done.

 3. *I think in the conclusion the authors should discuss the importance of testing the potentially meaningful differentiators in an independent dataset.*

 We agree. The discussion now includes: "ideally the results presented here should be confirmed in a new, independent sample."

 4. *It would also be very worthwhile in further studies to use individual participant data to determine the PPV of multiple predictors to engage in a diagnostic distinction.*

 That's an excellent idea. We have supplied individual participant data for the New Tics group as a supplementary file. 

 5. *If possible, a histogram exploring age and gender differences between the two groups would be helpful.*

 We do not have individual participant data for the FND-tic group (which came from 17 different publications), so we can't do this.
